# Network-based bioinformatics analysis of spatio-temporal RNA-Seq data reveals transcriptional programs underpinning normal and aberrant retinal development

**DOI:** 10.1186/s12864-016-2822-z

**Published:** 2016-08-31

**Authors:** Devi Krishna Priya Karunakaran, Sahar Al Seesi, Abdul Rouf Banday, Marybeth Baumgartner, Anouk Olthof, Christopher Lemoine, Ion I. Măndoiu, Rahul N. Kanadia

**Affiliations:** 1 Department of Physiology and Neurobiology, University of Connecticut, Storrs, CT 06269 USA; 2 Department of Computer Science and Engineering, University of Connecticut, Storrs, CT 06269 USA; 3 Utrecht University, 3508 TC Utrecht, The Netherlands

## Abstract

**Background:**

The retina as a model system with extensive information on genes involved in development/maintenance is of great value for investigations employing deep sequencing to capture transcriptome change over time. This in turn could enable us to find patterns in gene expression across time to reveal transition in biological processes.

**Methods:**

We developed a bioinformatics pipeline to categorize genes based on their differential expression and their alternative splicing status across time by binning genes based on their transcriptional kinetics. Genes within same bins were then leveraged to query gene annotation databases to discover molecular programs employed by the developing retina.

**Results:**

Using our pipeline on RNA-Seq data obtained from fractionated (nucleus/cytoplasm) developing retina at embryonic day (E) 16 and postnatal day (P) 0, we captured high-resolution as in the difference between the cytoplasm and the nucleus at the same developmental time. We found de novo transcription of genes whose transcripts were exclusively found in the nuclear transcriptome at P0. Further analysis showed that these genes enriched for functions that are known to be executed during postnatal development, thus showing that the P0 nuclear transcriptome is temporally ahead of that of its cytoplasm. We extended our strategy to perform temporal analysis comparing P0 data to either P21-Nrl-wildtype (WT) or P21-Nrl-knockout (KO) retinae, which predicted that the KO retina would have compromised vasculature. Indeed, histological manifestation of vasodilation has been reported at a later time point (P60).

**Conclusions:**

Thus, our approach was predictive of a phenotype before it presented histologically. Our strategy can be extended to investigating the development and/or disease progression of other tissue types.

**Electronic supplementary material:**

The online version of this article (doi:10.1186/s12864-016-2822-z) contains supplementary material, which is available to authorized users.

## Background

The retina has been the most accessible part of the developing central nervous system with a wealth of information on detailed birth order of its cell types and on many genes involved in executing specific programs such as cell cycle regulation, cell fate determination, and neuronal differentiation. However, a comprehensive gene regulatory network is still not achieved as gene-centric approach can only go so far. To address this issue, transcriptome capture to identify co-transcriptionally regulated genes across retinal development has previously been attempted and was of great value [1]. However, these efforts were hampered by the lack of depth of the captured transcriptome and lack of fractionation to gain higher resolution. Another concern was that at any given time the retina consists of different cell types with varied transcriptomes, which renders finding meaning from co-transcriptionally regulated genes difficult. We wanted to investigate whether higher depth of the captured transcriptome through RNA-Seq with minimal cross-compartment (nucleus-cytoplasm) normalization could resolve this issue.

Here we report analysis of RNA-Seq data from cytoplasmic and nuclear transcriptome of the developing retina. We show that combinatorial use of RNA-Seq with our custom bioinformatics strategy reveals the precise order of gene activation and transitions in processes during retinal development. Transition in gene expression was validated and resolved at the isoform level through our custom microarray. Importantly, we show proof of principle by extending our methodology to analyze RNA-Seq data from P21-Nrl-WT and KO retinae. Our approach which focuses on understanding the temporal progression in gene expression during normal/aberrant development can be extended to development and disease progression of other tissues.

## Methods

### Animal procedures

All experiments used CD1 mice from Charles River Laboratory, MA. All mice procedures were compliant with the protocols approved by the University of Connecticut’s Institutional Animal Care and Use Committee (IACUC).

### RNA fractionation

Retinae were dissected from E16 embryos and P0 pups followed by fractionation protocol as described previously [2]. Once the fractions were obtained, Trizol (Invitrogen, CA, cat # 15596-026) was used as per the manufacturer’s instructions.

### Library preparation for deep sequencing

After the total RNA was prepared from the two fractions, ribosomal RNA (rRNA) was removed using Ribozero Ribosomal RNA removal kit (Epicenter, WI, cat # RZH1046) by following the manufacturer’s instructions. The removal of rRNA was confirmed by gel electrophoresis and was used for RNA-Seq library preparation. RNA-Seq library was prepared using Script-seq mRNA seq library preparation kit (Cambio, UK, cat # SS10906). The library was deep sequenced in multiple runs using Illumina Hi-Seq 2000 platform at the University of Connecticut Health Center Deep sequencing core facility. P21 Nrl- WT and KO RNA-Seq data was shared with us by Dr. Anand Swaroop; National Eye Institute [3].

### RNA-Seq analysis

#### CD1 reference creation

The transcriptome captured by deep sequencing was obtained as short paired-end reads. We analyzed the RNA-Seq data from each sample through riboPicker [4], an algorithm to identify reads derived from rRNA, which showed minimal (0.02–0.34 %) rRNA reads (Table 1). Next, reads were mapped to the mouse genome and the transcriptome to create a reference. The mouse genome sequence (mm10, NCBI build 38) was downloaded from UCSC database [5, 6] together with the GTF for the Ensembl transcript library (release 68) (http://genome.ucsc.edu). The paired-end reads from E16 cytoplasmic extract (CE), P0CE and P0 nuclear extract (NE) were mapped separately to the mm10 genome and the transcript sequences extracted according to the Ensembl transcript library coordinates. Mapping was done using bowtie [7] and allowed for three mismatches in seed-length of 30 bases. For each sample, the two sets of read alignments (genome and transcriptome) were merged together using the HardMerge tool from the NGSTools suite [8]. HardMerge discards reads that align at multiple locations in the genome or transcriptome as well as reads that align uniquely to each but at discordant locations. This initial mapping was used to perform mismatch analysis with another tool in the NGSTools suite (Additional file 1: Figure S1). Accordingly, the first 6 and last 32 bases from each read were trimmed. The trimmed reads (62 bp) were remapped using the aforementioned mapping parameters to the genome and transcriptome, and were once again merged using the HardMerge rules. Since the RNA-Seq was performed on retinal RNA extracted from CD1 strain mice, the resulting alignments from all three samples were pooled together and used to call Single Nucleotide Variations (SNVs) using SNVQ [8]. A CD1 reference genome sequence was created by modifying the mm10 reference to reflect the inferred SNVs. Transcript sequences were extracted from this CD1 genome based on Ensembl 68 annotations.

**Table 1:** Read mapping statistics and rRNA levels in the E16CE, P0CE, and P0NE samples

Sample	Percentage of transcriptome mapped read pairs	Percentage of rRNA reads	# mapped bases in Gb
E16CE	61.26 %	0.34 %	7.54
P0CE	52.85 %	0.19 %	7.69
P0NE	25.42 %	0.03 %	4.02

### Gene expression analysis

E16CE, P0CE and P0NE reads were mapped against the CD1 Ensembl 68 transcriptome reference. The P21 WT and KO single end reads were mapped against the C57BL6 reference transcriptome based on Ensembl version 68. Mapping was done using bowtie and allowed for one mismatch in an alignment seed of 30 bases. Gene expression levels were estimated using IsoEM [9], an expectation-maximization algorithm that estimates isoform frequency from single and paired RNA-Seq reads. IsoEM exploits read disambiguation information provided by the distribution of insert sizes generated during sequencing library preparation, and takes advantage of base quality scores, strand, and read pairing information. Isoform expression is reported as Fragment per Kilobase per million mapped reads (FPKM) units and gene expression is the sum of FPKM of its constituent isoforms. For gene differential expression, two methods were run, GFOLD [10] and Fisher’s exact test with house-keeping gene normalization as in [11]. Gapdh was used as the housekeeping gene for this analysis. Genes were called differentially expressed if they showed ≥2 fold expression in one sample by both methods. GFOLD was run on the CD1 transcriptome aligned reads, with default parameters and a p-value of 0.01. Fisher’s exact test was run on estimated number of reads mapped per kilobase of gene length (calculated from IsoEM estimated FPKM values). Similar to GFOLD, a p-value of 0.01 was used for Fisher’s exact test.

### Binning strategy

**Fig. 1:**
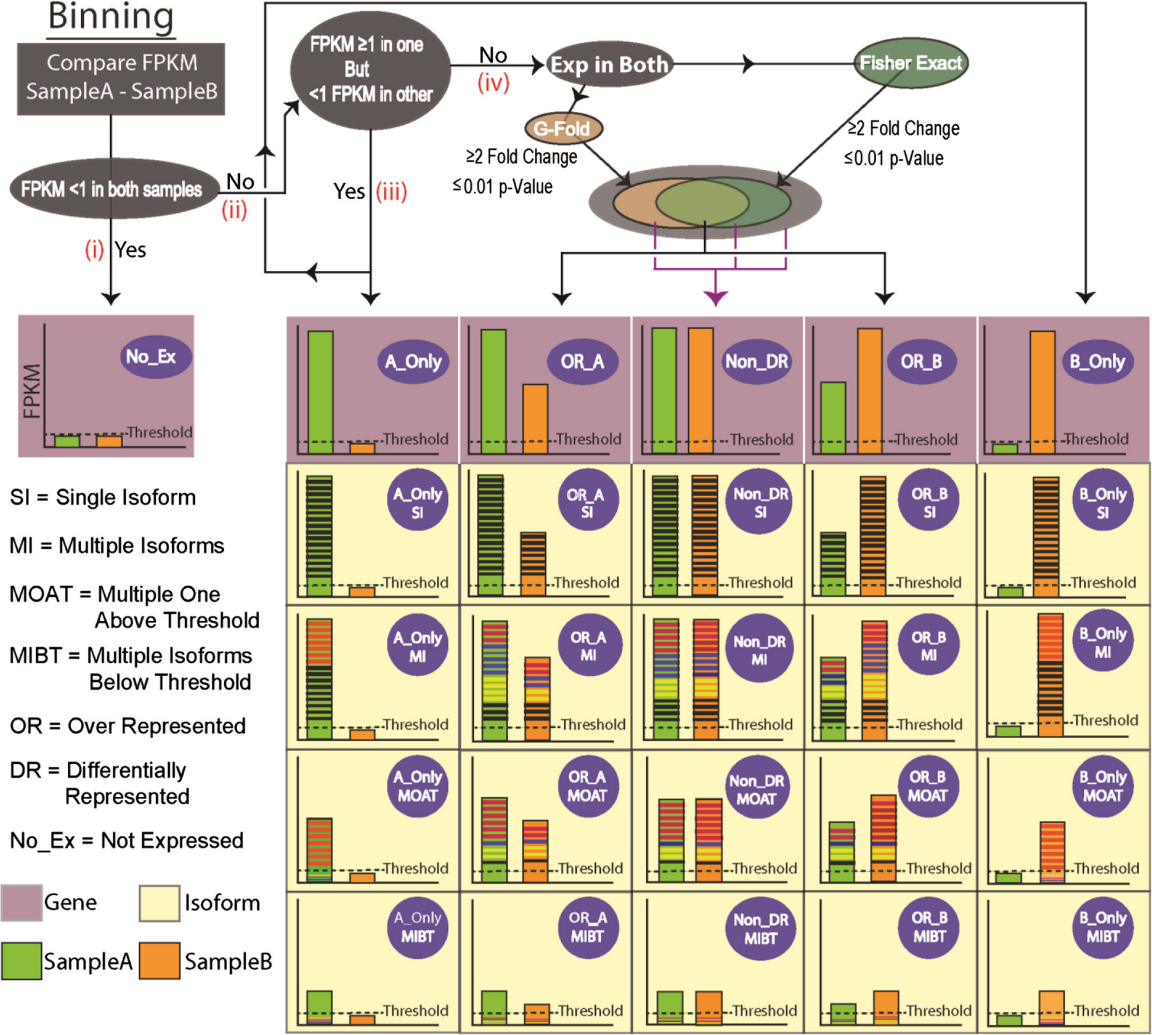
Binning strategy for RNA-Seq data. Binning protocol shown here is for two theoretical samples, A and B. Schematic on the top shows the different steps in the binning protocol and the outcomes are shown as bar graphs underneath (*purple boxes*). In the bar graph FPKM units are shown on the y-axis and the gene is represented as a bar in green (sample A) and orange (sample B). Colored lines within the bar represent the constituent isoforms (*Yellow boxes*). The dashed line represents the threshold (1 FPKM) of gene expression

Samples were analyzed in pairs, and genes were classified based on their expression levels (expressed vs. not expressed), differential gene expression status, and the number of expressed isoforms. 1 FPKM was set as threshold for expression. Genes were then classified into one of the following bins (Fig. 1) based on yes/no calls. Firstly, genes with expression level less than 1 FPKM in both compared samples are placed in the not expressed (No_Ex) bin (Fig. 1i). The rest of the genes, which were expressed in at least one sample, were further categorized into the following bins (Fig. 1ii). Genes expressed exclusively in one sample were placed in one of the ONLY bins (Fig. 1iii). Differential expression calls were made for genes expressed in both samples (Fig. 1iv). If a gene passed GFOLD and Fisher’s test, then it was placed in the over represented (OR) bin. Genes, which did not pass both or either of these tests, were placed in the non-differentially represented (non_DR) bin. Bins of expressed genes were subcategorized based on the alternative splicing status of the genes. This categorization included single and multiple isoform bins (SI, MI). There were genes with multiple isoforms that were individually below threshold, but the sum of FPKM values of these isoforms is above threshold. These were placed in the multiple isoforms below threshold (MIBT) bin. Similarly, genes expressed with multiple isoforms where only one isoform was above threshold were placed in the multiple isoforms one above threshold (MOAT) bin.

### Functional annotation analysis

**Fig. 2:**
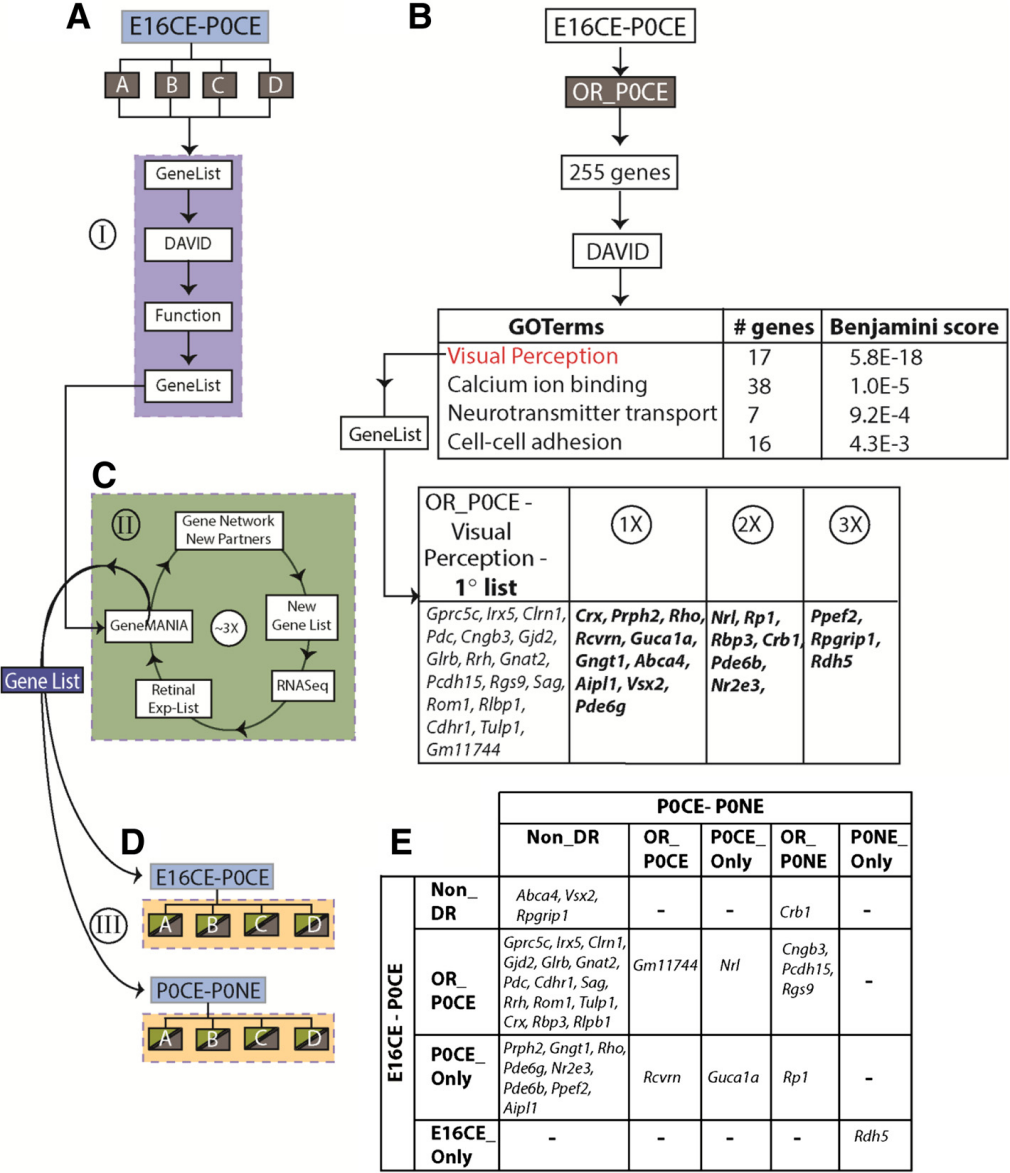
Custom bioinformatics pipeline revealed transition in biological processes. **a** E16CE-P0CE comparison is shown with its bins as boxes that were used to extract gene list for DAVID analysis and the GO terms for functions enriched by these lists were curated (Detailed list of GO terms in Additional file 2: Table S1.1, S1.2). This process is represented by the roman number I. **b** An example to show the output of Part I, where OR_P0CE bin was chosen from E16CE-P0CE comparison. The genes that enriched for a function in each bin in **a** were then subjected to pipeline shown in **c** (II), which starts with gene list entry to GeneMANIA followed by (*arrow going up*) identification of new partners. (*Right*) Output of the pipeline in **c** (II), where the primary gene list (17 genes) that enriched for “Visual Perception” function is shown in the first column. The three iterations of the pipeline in part II are denoted as 1X, 2X and 3X. **d** (III). Genes in the final list were assigned to their bins in E16CE-P0CE and P0CE-P0NE comparisons. **e** Output for **d** (III) with rows showing distribution of genes in bins from E16CE–P0CE comparison and the columns reflecting genes in bins from P0CE-P0NE comparison

Genes belonging to each bin were analyzed for enrichment of individual GO terms to find whether co-transcriptionally regulated genes had overlapping functions using the Database for Annotation, Visualization and Integrated Discovery, DAVID (Fig. 2a-i) [12, 13]. Default parameters (≤0.05 Benjamini score) were used for all analyses. The gene lists enriching for GO terms were run through another online tool called GeneMANIA to identify potential partners [14]. First, we took the gene list underlying a biological process identified by DAVID and used it as bait in GeneMANIA (Fig. 2c-II), which identified potential partners for genes from the primary list. The potential partners identified by GeneMANIA are based on published literature and publicly available databases, which could introduce a partner that is relevant in another tissue, but might not be expressed in the retina. To eliminate such genes, we selected only those genes that were expressed in our RNA-Seq data. Subsequently, this short list of genes was added to the primary list to generate a secondary list, which was used again as bait in GeneMANIA. This process was repeated until convergence, which was reached after three iterations (Fig. 2c-II).

### Microarray design

We designed a custom Affymetrix microarray to en masse interrogate the presence/absence of unique exon/exon junctions in isoforms expressed in the RNA-Seq data. After mapping the RNA-Seq reads from the three samples (E16-CE, P0-CE and P0-NE) to the Ensembl 68 transcripts and running IsoEM to estimate the FPKM values for each of the three samples, genes expressed in any of the three samples were selected. Exon-exon junctions that are unique among expressed transcripts in genes that have more than one expressed transcript were selected (junctions with flanking sequences of length ≤12 bases were eliminated). As a result, we included probes for 28,574 unique junctions from 11,923 transcripts on the custom Affymetrix chip.

### Custom affymetrix data analysis

Cytoplasmic RNA (1 μg) was prepared from retinae harvested from E12, E16, E18 embryos and P0, P4, P10 and P25 and processed at Yale Center for Genome Analysis. Expression levels of probe targets were computed from the raw intensity values using the Robust Multichip Average (RMA) algorithm [15, 16], which was performed with the affy R package [17]. Subsequently, the data were processed through Gene Expression Similarity Investigation Suite (Genesis) (v1.7.6) for k-means clustering [18]. Here, we ran 500 iterations to generate a total of 10 clusters that fell into three categories based on expression kinetics. These trends were defined as embryonic, postnatal, and embryonic + postnatal. The gene lists belonging to each cluster were separately analyzed for functional enrichment using DAVID.

## Results

### RNA-Seq of fractionated retina

RNA for deep sequencing was obtained from retinae from E16 as it is the midpoint in embryonic development and P0 as it is a major transition in development. RNA-Seq was performed on rRNA depleted RNA captured from the cytoplasmic extract (CE) of E16 and P0 along with nuclear extract (NE) of P0. The rationale was that by comparing the CE across development, we would capture mRNA that were most likely translated into proteins, which in turn would reveal transitions in biological processes during the retinal development. Also, comparing the cytoplasmic transcriptome minimizes the contamination of unspliced transcripts contributed by the nuclear fraction, which might spike the FPKM units of isoforms and in turn the gene expression. On the other hand, comparison of P0CE to P0NE would reveal transcriptome dynamics within the P0 time point. The two-way comparison (time and fraction) would capture change in transcription kinetics with three distinct sets of transcripts: transcripts in both fractions; transcripts exclusively in the CE; and transcripts exclusively in the NE. Overall, the transcriptome captured by RNA deep sequencing was obtained as 99.28, 117.38, and 127.46 million paired-end reads from E16CE, P0CE, and P0NE, respectively. An important decision for RNA-Seq analysis is setting the threshold for gene expression, which in the field ranges from 0.3–1.0 FPKM [19]. This range suggests that the threshold for any dataset must be vetted through empirical evidence. Thus, we interrogated a range of FPKM values to set the threshold for gene expression in the retina. We found that 1 FPKM was the appropriate value, as genes with known retinal expression were above this threshold, hence considered expressed. In contrast, skeletal muscle-specific genes were below 1 FPKM, and were considered not expressed in the retina (Table 2). While the low-level expression (<1 FPKM) of skeletal muscle-specific genes might have a yet-to-be-identified biological function in the retina, we reasoned that in the absence of any literature support it would be safe to consider them as not expressed, to ensure high specificity of our analysis, at the cost of possible slight loss in sensitivity. Once the threshold was set, the reads were then subjected to our custom bioinformatics pipeline as described in materials and methods. The output of mapping and gene expression quantification was reported in Fragment per Kilobase per million mapped reads (FPKM) units.

**Table 2:** Skeletal muscle-specific genes expression. The table shows FPKM values of skeletal muscle-specific genes in E16CE, P0CE and P0NE samples

Skeletal-muscle genes	E16CE FPKM	P0CE FPKM	P0NE FPKM
Tnnt3	0.10	0.11	0.00
Tnnt1	0.94	0.50	0.50
Tnni3k	0.00	0.02	0.10
Tnni2	0.30	0.21	0.20
Tnni1	0.10	0.90	0.44
Tnnc2	0.00	0.00	0.10
Tnnc1	0.63	0.95	0.91
Tnn	0.00	0.00	0.10

### Validation of RNA-Seq data

**Fig. 3:**
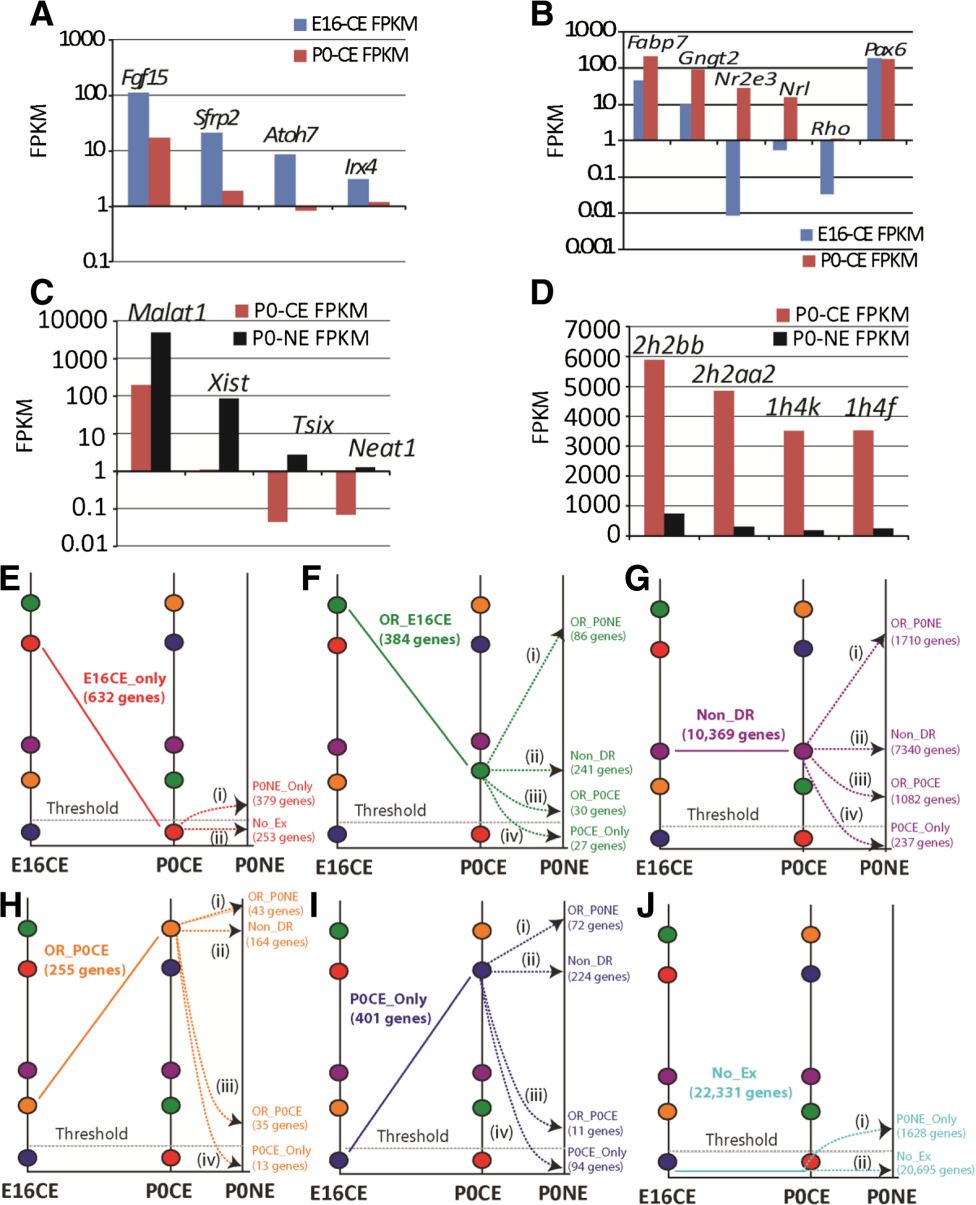
Validation of high-resolution transcriptome by RNA-Seq. **a**–**d** Expression of genes with established expression kinetics including **a** Fgf15, Sfrp2, Atoh7, Irx4, **b** Fabp7, Gngt2, Nr2e3, Nrl, Rho, Pax6, **c** Malat1, Xist, Tsix, Neat1, **d** Hist2h2bb, Hist2h2aa2, Hist1h4k and Hist1h4f shown as bar graph with FPKM in y-axis (log scale for **a**–**c**) between E16CE (*blue*), P0CE (*red*), and P0NE (*black*). **e**–**j** Combined E16CE-P0CE and P0CE-P0NE high-resolution transcription kinetics, E16CE_Only (**e**), OR_E16CE (**f**), Non_DR (**g**), OR_P0CE (**h**), P0CE_Only (**i**) and No_Ex (**j**). OR_E16CE - Over represented in E16CE; OR_P0CE – Over represented in P0CE; Non_DR – Non-differentially represented

We used genes with established expression kinetics to objectively assess the sensitivity of RNA sequencing [20]. For example, *Fgf15*, *Sfrp2*, *Atoh7* and *Irx4* are known to have higher expression levels at E16 than at P0, which was reflected in E16CE compared to P0CE data (Fig. 3a) [1, 21–25]. Likewise, expression of *Fabp7*, *Gngt2*, *Nr2e3*, *Nrl*, and *Rho* was as predicted in that it was higher in P0CE compared to E16CE (Fig. 3b) [1, 26–32]. Finally, *Pax6* showed little variation between E16CE and P0CE (Fig. 3b), which was also as expected [33, 34]. The transcriptional kinetics of some of these genes was independently validated by qPCR analysis across retinal development (E14, E16, E18, P0, P2, P4, P10, P25), thereby confirming the robustness of both RNA-Seq data and the bioinformatics approach used to assign expression and binning [2].

We also used the same paradigm of genes with established expression kinetics to determine the level of cross-contamination between P0CE and P0NE RNA. To determine the level of nuclear RNA contaminating the cytoplasmic extract, we checked the expression of genes whose transcripts are predominantly nuclear, such as *Xist*, *Malat1*, *Tsix* and *Neat1* [2, 35–37]. Indeed, for all four genes, the FPKM values were significantly higher in the NE compared to the CE (Fig. 3c). Determining the level of cytoplasmic RNA contamination in the nuclear extract presented a unique challenge as the majority of the RNA in the CE would be expected to be in the NE. For this, we examined expression of replication-dependent histone genes, as they are intronless and are known to be efficiently exported to the cytoplasm [38]. Indeed, histone genes have higher FPKM in the CE than the NE (Fig. 3d). Furthermore, replication-dependent histone genes also serve to account for genomic DNA contamination. These genes lack introns and do not require splicing, so histone genomic DNA would be read as histone mRNA and inflate FPKM values in the NE, which was not the case (Fig. 3d), thus confirming minimal genomic contamination. Finally, genomic DNA contamination would result in FPKM value >0 for all genes; however, we observed 0 FPKM in the NE for a large number (804) of genes. In all, these controls suggest that there was minimal genomic DNA contamination in our fractionated NE RNA-Seq data.

### RNA-seq revealed high-resolution transcription kinetics

We wanted to test the fidelity of our approach in capturing in vivo kinetics and the identification of co-transcriptionally regulated genes. Inherent to our binning strategy is identification of co-transcriptionally regulated genes. Therefore, the aforementioned binning strategy (Fig. 1) was employed for the E16CE vs. P0CE comparison and the P0CE vs. P0NE comparison to extract the transcription kinetics. For example, genes in the E16CE_Only bin had FPKM below threshold in P0CE. This suggests that transcription of these genes was initiated at/before E16 and was downregulated after E16 or just before P0. Genes in the P0CE_Only bin were transcribed after E16, but before P0. Genes in the OR_E16CE bin suggest that their transcription was initiated at/before E16 and downregulated after E16, but before P0 such that their FPKM was not below threshold in P0CE. Overall, 12,041 gene were expressed (Additional file 1: Figure S2) of which 10,369 were non-differentially represented (Non_DR) between E16CE and P0CE (Additional file 2: Table S1.1, S1.2, Additional file 1: Figure S2). Further analysis of alternative splicing status showed that genes in Non_DR bin were alternatively spliced at a higher level (42 %) compared to those undergoing transcriptional change (37 %, Additional file 1: Figure S2). Likewise, the binning strategy was employed with the P0CE-P0NE comparison, which showed an increase in the number of expressed genes, which was mostly accounted for by the 2007 genes in the P0NE_Only bin (Additional file 1: Figure S2). Investigation of the alternative splicing status showed that genes over represented in P0NE (OR_P0NE) employed the highest degree of alternative splicing (68 %) compared to other bins including OR_P0CE (36 %) and Non-DR (52 %) (Additional file 1: Figure S2). Next we combined E16CE-P0CE transcription kinetics with those observed in P0CE-P0NE transcription kinetics. Briefly, we took genes in a bin from the E16CE-P0CE comparison and interrogated their distribution in the different bins in the P0CE-P0NE analysis, which yielded high-resolution transcription kinetics. The term “high-resolution transcription kinetics” encapsulates both temporal and detection sensitivity. For example, amongst the genes in the E16CE_Only (632) and not expressed (No_Ex, 22,331) bins, 379 and 1628 genes were detected above threshold in the P0NE_Only bin, respectively (Fig. 3e and j). Similarly, 86 genes from the 384 genes in the OR_E16CE bin and 1710 genes from the 10,369 genes in the Non_DR bin were upregulated in the P0NE compared to the P0CE (Fig. 3f and g). In contrast, 35 genes of the 255 genes in the OR_P0CE bin were downregulated in P0NE compared to P0CE, while FPKM for 13 genes was below threshold (Fig. 3h). Likewise, 11 genes of 401 genes found in P0CE_Only in the E16CE-P0CE comparison were downregulated in P0NE compared to CE and 94 genes had FPKM below threshold (Fig. 3i). Overall, there were 2007 genes with transcripts exclusively in P0NE, of which 1084 were protein coding genes, 582 were Gm clones, 214 were Riken clones, and the rest were non-coding RNA genes (Table 3).

**Table 3:** Distribution of 2007 transcripts in P0NE_Only sample

Type of RNA	# genes
Protein coding genes	1084
Gm clones	582
Riken clones	214
Miscellaneous/Anti-sense/non-coding/rRNA	35
Pseudogenes	33
snoRNA	21
microRNA	27
lincRNA	5
snRNA	6

### Transcriptionally coupled genes revealed molecular programs in the developing retina

**Fig. 4:**
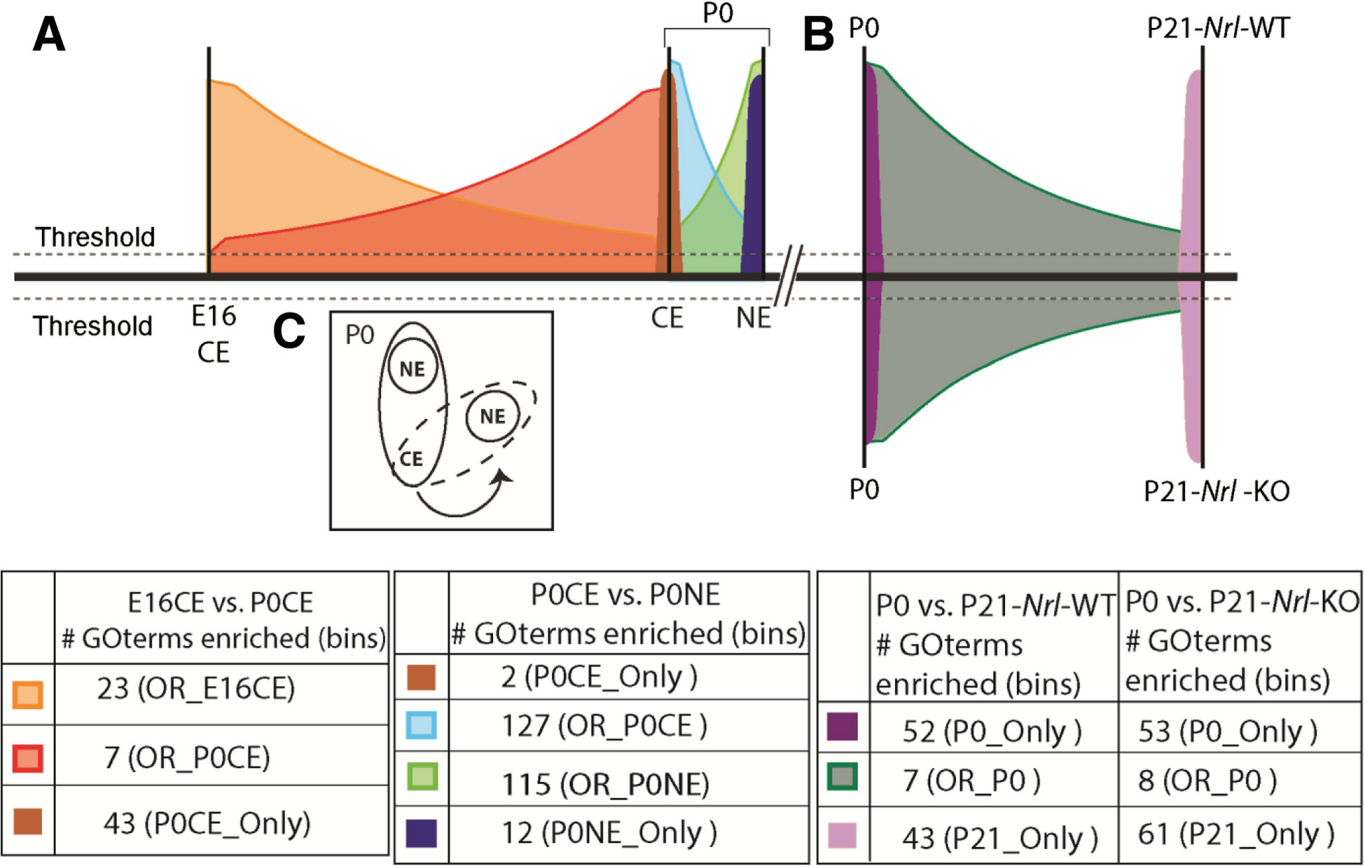
RNA-Seq revealed progression of biological programs across normal and aberrant retinal development. **a** Schematized representation of kinetics of the molecular programs identified by RNA-Seq (E16CE-P0CE- P0NE) is represented with different colors and the shapes represent the gene expression kinetics. **b** Extension of our temporal analysis strategy to P21-Nrl-WT and P21-Nrl-KO by comparing them to P0 (P0CE + P0NE) revealed molecular programs in normal development (P21-Nrl-WT) and unique programs in aberrant development (P21-Nrl-KO). **c** Schematic representation of a cell at P0, where the cytoplasm and the nucleus are temporally synchronized and the cell with dotted line shows that the nuclear transcriptome is shifted forward temporally compared to that of the cytoplasm

Our objective here was to employ RNA-Seq to find transcriptionally coupled genes so that we could leverage them to discover molecular programs being employed during retinal development. For this, genes were subjected to DAVID analysis (Fig. 2a-i). Interestingly, our first submission of genes (632; Fig. 3e) in the E16CE_Only bin to DAVID did not enrich for any statistically significant (Benjamini <0.05) functions (Fig. 2b). However, other bins with fewer or more genes than in E16CE_only yielded many functions (Fig. 4, Additional file 3: Table S2.1). For example, genes in the OR_P0CE bin enriched for 7 functions of which the top hit was “visual perception” (Fig. 2b, Additional file 3: Table S2.1), showing that this function was initiated just before birth. This showed that transcriptionally coupled genes could inform biological processes that were executed at that developmental timepoint.

One caveat to our binning strategy was that while it grouped genes based on transcription kinetics, it may have separated genes participating in a common biological process (identified by DAVID) into different bins. This in turn would prevent extraction of the expression kinetics of genes known to participate in executing a biological process of interest. To address this issue, we devised an iterative approach using GeneMANIA (Fig. 2c-II) to identify genes participating in a common biological process from the different bins. For example, 17 genes in OR_P0CE genes (E16CE-P0CE) that enriched for visual perception by DAVID were used as bait in GeneMANIA, followed by the aforementioned iterations (Fig. 2c; 1x-3x) to generate a final list of 36 genes (Fig. 2c, Right). Subsequently, each gene was assigned to its respective bin in both E16CE-P0CE and P0CE-P0NE comparisons (Fig. 2d-III). Redistribution of the genes into their respective bins is shown in Fig. 2e. For visual perception, the transcription of *Rdh5*, which converts all-trans retinal to 11-cis retinal [39] was initiated at/before E16, shut down just before birth and was initiated again at birth as we find it in P0NE_Only bin in the P0CE-P0NE comparison (Fig. 2e). In contrast, *Crb1, Cngb3, Pcdh15* and *Rgs9*, which play a role in photo transduction [40, 41] and structural support/maintenance of photoreceptors [42, 43], were transcribed at/before E16 and upregulated at P0 as they were over-represented in P0NE in P0CE-P0NE (Fig. 2e). *Rp1*, which is a photoreceptor-specific microtubule-associated protein [44], was the only gene that was transcribed at P0 (P0CE_Only in E16CE-P0CE) that continued to be upregulated as it was over-represented in P0NE in P0CE-P0NE (Fig. 2e). Transcription of *Guca1a* was initiated between E16 and P0 (P0CE_Only in E16CE-P0CE), except it was turned off before P0 (P0CE_Only in P0CE-P0NE) (Fig. 2e). Through this method we were able to deconstruct the precise activation of genes involved in many aspects of vision acquisition/phototransduction during embryonic development.

The same analysis was performed on all the bins in P0CE-P0NE comparison. Specifically, genes in the OR_P0NE bin showed enrichment for 120 GO terms of which one of them was “synapse” (Additional file 3: Table S2.2). This functional enrichment agrees with studies showing that synaptogenesis occurs postnatally in the rodent retina [45]. Further analysis of genes underlying the GOterm “synapse” showed transcription initiation of the AMPA receptor subunit genes including, *Gria1*, *Gria2* and *Gria4* before/at E16 (Non_DR in E16CE-P0CE) (Additional file 1: Figure S3). Similarly *Grik2*, which encodes for a subunit of the ionotropic kainate receptor, was also initiated before/at E16 (Additional file 1: Figure S3). *Gad2*, which is necessary for the production of the inhibitory neurotransmitter GABA, was transcribed before E16, while *Gad1* transcription was initiated after E16 prior to birth (Additional file 1: Figure S3). Overall, genes involved in formation of the presynaptic activity were activated mostly during embryonic development (Additional file 1: Figure S3). In contrast, genes involved in postsynaptic activity had overlapping transcriptional activation with a subset of genes (*Grid2, Grid1, Grik5, Grin3a, Ryr2* and *Shank2*) that were specifically activated in P0NE (Additional file 1: Figure S3). Finally, employing the same analysis for genes in P0NE_Only, which reflected *de novo* transcription, enriched for 14 GO terms, of which voltage-gated calcium ion channel activity was one of the top hits (Additional file 3: Table S2.2). Again, this enrichment did agree with previous studies where it has been shown that calcium channel activity is crucial for the construction of functional synapses that occurs postnatally [45].

### Extending the analysis to other time points through the custom microarray

**Fig. 5:**
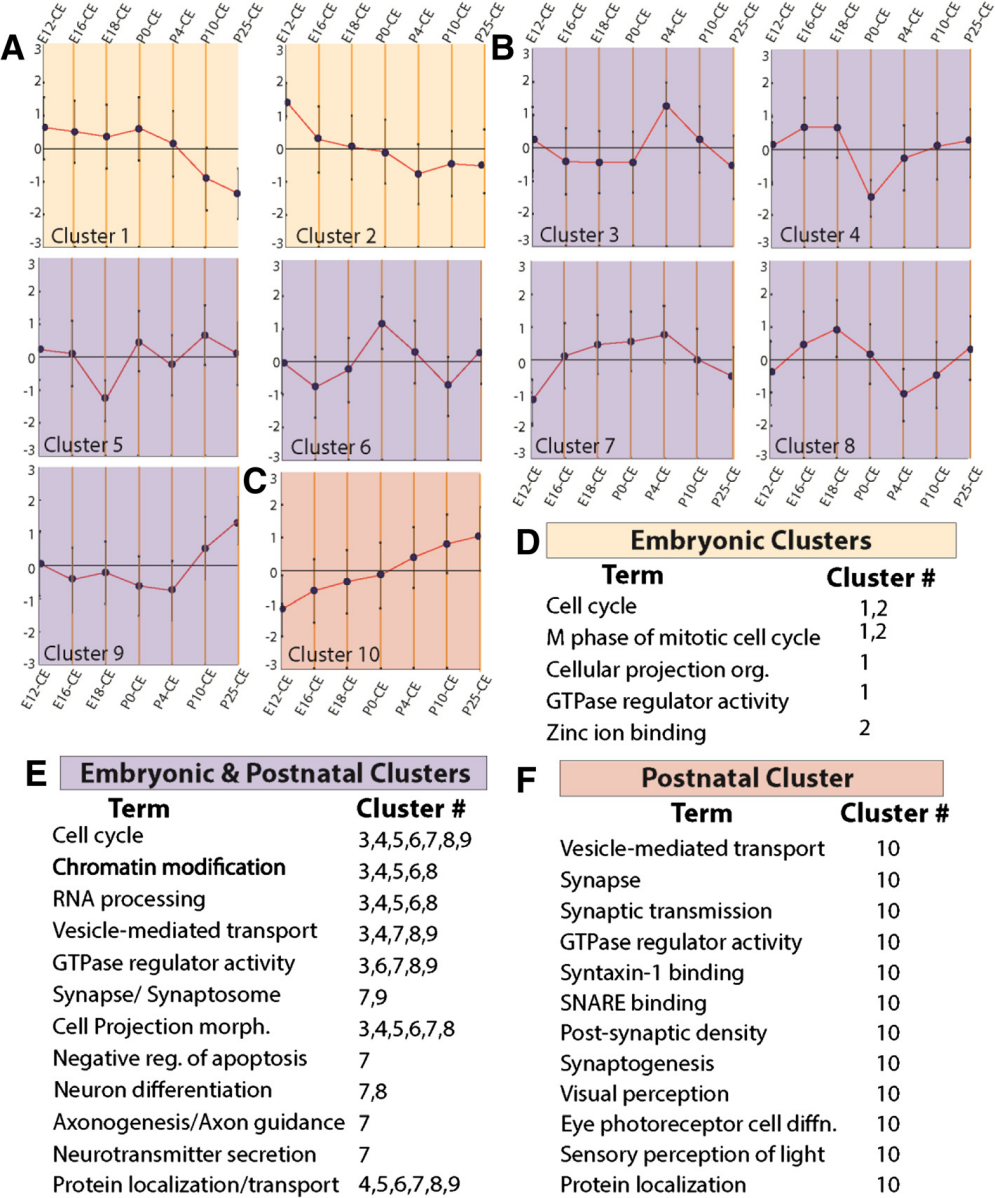
Custom microarray revealed isoform/gene expression coherence and validated RNA-Seq. **a**–**c**. Shown here is a centroid view of K-means clusters of isoform-specific probes across retinal development (Clusters given in Additional file 4: Table S3). The y-axis shows arbitrary units (−3 to 3) of expression and the developmental time is shown on top and bottom. **d**–**f** Selected GO terms enriched by DAVID analysis for genes in clusters (**a**-**c**)

To confirm our RNA-Seq findings and extend our analysis across retinal development, we leveraged our RNA-Seq data to design a custom microarray. The array was designed to en masse validate isoform kinetics by assaying for unique exon-exon junctions of a subset of genes across retinal development. The junctions were selected based on the following criteria: 1) gene must have more than one isoform expressed in the RNA-Seq data; and 2) An exon-exon junction must be unique such that it is not found more than once in all of the isoforms for that gene in the Ensembl database. In all, the microarray had 28,575 probes for 5581 genes and was employed to interrogate expression of these isoforms in the cytoplasmic transcriptomes of E12, E16, E18, P0, P4, P10 and P25 retinae. Data obtained were subjected to K-means clustering with the Genesis software, which was set to generate 10 clusters (Additional file 4: Table S3). Based on the overall patterns across time, the clusters were organized into three groups: embryonic, embryonic + postnatal and postnatal clusters (Fig. 5a-c), subsequently referred to as clusters 1, 2, and 3, respectively.

Next, we applied DAVID analysis to each cluster and found that embryonic clusters (Clusters 1 and 2) enriched for functions such as cell cycle regulation and cell projection organization (Fig. 5d). For the embryonic + postnatal clusters (Clusters 3–9), the functional GO terms that were enriched were those required for cell cycle regulation and terminal differentiation of neurons, such as vesicle-mediated transport, synapse formation, negative regulation of apoptosis, and axon guidance (Fig. 5e). Finally, the sole postnatal cluster (Cluster 10) was the only one that enriched for functions such as visual perception, photoreceptor cell differentiation and sensory perception of light (Fig. 5f). In all, the isoform-specific microarray confirmed RNA-Seq findings and further revealed the complexity of alternative splicing employed by the developing retina.

### Comparison with other analysis methods

Our analysis pipeline is characterized by two main features, namely the temporal nature of the analysis and the binning strategy that allow us to do the functional analysis on genes with very specific expression kinetics. The current norm for next generation sequencing based functional analysis in the literature is done based on simple differential expression (DE) analysis, where the whole list of DE genes is fed into DAVID, or a similar functional annotation analysis platform. The list of DE genes can sometimes be too large, exceeding the input size limit of the functional analysis tool. To overcome this problem, a sub-list is sometimes selected based on prior knowledge of the gene functions [46]. Most analysis are also done in a static manner, where samples represent two conditions at the same time point [3]. In this section, we studied the effect of binning and the temporal analysis on the result, by varying the analysis strategy. We first applied our analysis strategy to RNA-Seq data from P21-*Nrl*-WT and P21-*Nrl*-KO (Courtesy Dr. Anand Swaroop; NEI) [3]. Then we applied three variants of the analysis pipeline, listed in Table 4, to the same data, and compared the results.

**Table 4:**
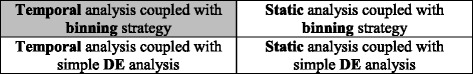
The table shows our analysis strategy (highlighted in grey) and the three other analysis variants compared.

### Temporal analysis combined with static analysis of *Nrl* WT and KO RNA-Seq is more informative

The loss of *Nrl* results in cell-fate switch from rod to cone photoreceptors [47]. This made *Nrl*-KO an ideal system to test our hypothesis that temporal comparison would yield more information than the static analysis. First we performed static comparison between P21-*Nrl*-WT and P21-*Nrl*-KO data (Additional file 1: Figure S4, Additional file 5: Table S4.1), similar to the one previously reported [47]. The objective of transcriptomics analysis of wild-type and knockout tissue is to find genes undergoing change to reveal the resulting biological change in the absence of that gene. Surprisingly, DAVID analysis of genes undergoing dynamic changes in gene expression in static comparison of P21-*Nrl*-WT and KO enriched for a couple of generic functions that did not give any meaning in terms of the knockout phenotype (Additional file 3: Table S2.5).

**Fig. 6:**
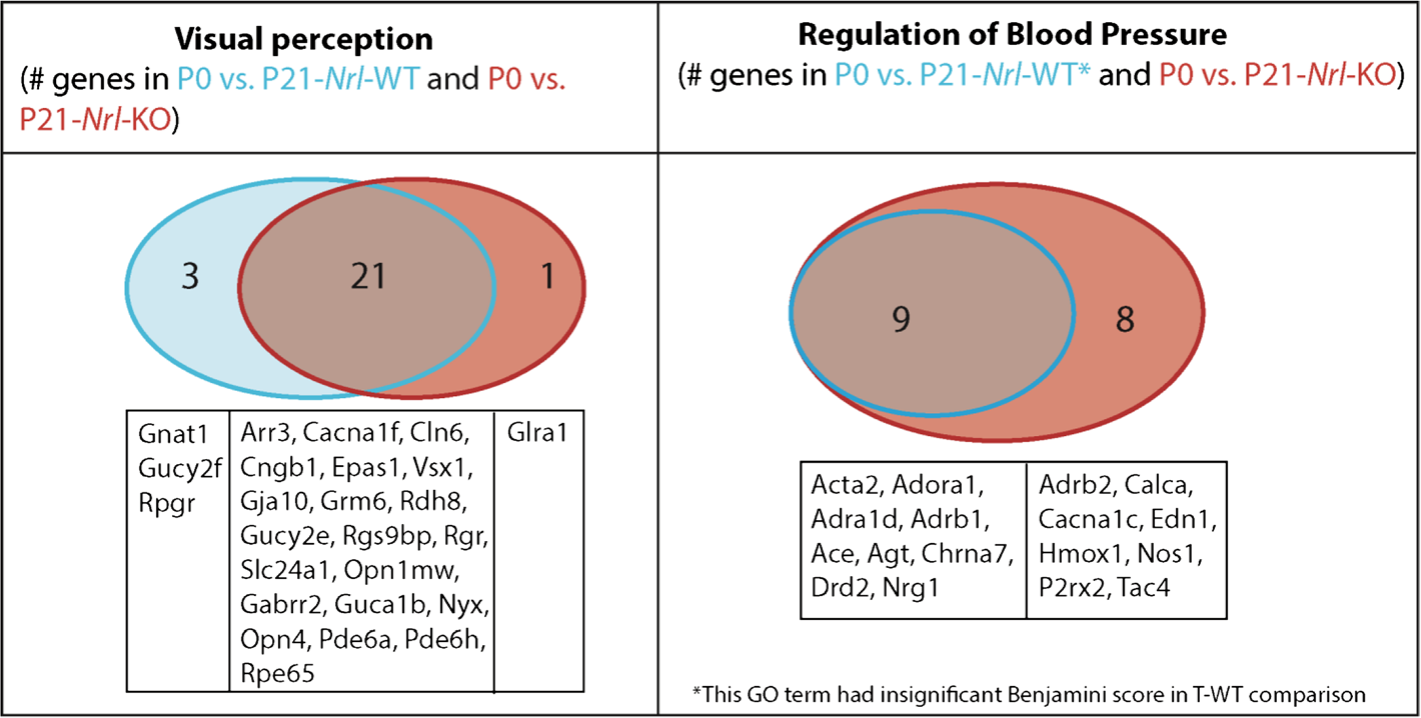
Temporal comparison of P21-Nrl-WT and P21-Nrl-KO to P0. Shown is an example of common and unique GO terms identified in the P21_only bin in both P0 vs. P21-*Nrl*-WT (P21WT_Only) and P0 vs. P21-*Nrl*-KO (P21KO_Only) comparisons and the genes underlying them. (Left) common function (visual perception); (Right) Unique to P0 vs. P21-*Nrl*-KO (regulation of blood pressure) (Detailed list of GO terms in Additional file 2: Table S1.3, S1.4)

Our RNA-Seq analysis of data from E16CE to P0CE and P0CE to P0NE comparisons showed that comparison across time (∆/time) was crucial in revealing biologically relevant meaning from co-transcriptionally regulated genes. Therefore, we introduced the variable of time by comparing P21-*Nrl*-WT and P21-*Nrl*-KO data separately to our P0 data (P0CE + P0NE) (Additional file 1: Figure S4, Additional file 5: Table S4.2, S4.3). The rationale was that ∆/time would reveal unique sets of gene in P0 vs. P21-*Nrl*-KO comparison, which in turn would reveal changes in the biological processes. In both comparisons, there were several bins with co-transcriptionally regulated genes (Additional file 1: Figure S4), which is not surprising, considering the major developmental shift from newly born developing retinae at P0 to fully functional P21 retinae. Also, DAVID analysis yielded significant GO terms for all the bins in the temporal analysis (Additional file 3: Table S2.3, S2.4). Moreover, there were many common functions in P21_Only bin (P21WT_Only and P21KO_Only) in both analyses. The most relevant issue was to ascertain the overlap in the identity of genes for the same process in the P21_Only bin in both sets of comparisons. For example, one of the common function in P21_Only bin was “visual perception”. Interrogating the genes underlying the GO term “visual perception” in these bins, there were 21 genes common to both P0-P21WT and P0-P21KO analysis. However, 3 genes (*Gnat1, Gucy2f, Rpgr*) were only found in the P0-P21WT comparison, and one gene (*Glra1*) was specific to the P0-P21-KO comparison (Fig. 6, Left). The three genes unique to WT comparison are known to operate specifically in rod photoreceptors, which were of course absent in the *Nrl*-KO retina [48–50]. On the other hand, *Glra1* is an important gene for cone-bipolar cells, which might be undergoing adaptive changes in the *Nrl*-KO retina [51]. The GO terms enriched by genes in P21_Only bin in the P0 vs. P21-*Nrl*-KO comparison were also informative. One of the functions enriched in DAVID was “regulation of blood pressure” (Fig. 6, right). Analysis of function of the genes underlying this enrichment revealed that most of them were engaged in vasodilation, suggesting that the *Nrl*-KO retina was undergoing vasculature restructuring/dilation as a secondary effect of the cell-fate switch. Moreover, a recent report showed that the *Nrl*-KO retina develops dilated retinal blood vessels and leakage at P60 [52]. This showed that our gene expression/binning strategy captured the molecular signature at P21 for a phenotype that manifests histologically at P60.

### Binning vs. DE based analysis of *Nrl* WT and KO data

Here we have introduced a new strategy for RNA-Seq data analysis. To understand the advantages afforded by this approach we compared our binning method to the more commonly used DE method. First we analysed the static comparison between *Nrl*-WT vs. *Nrl*-KO data by the binning method and the DE-based method. Here we found that the DE-based method showed a large number (1116) of genes with differential expression (Additional file 6: Table S5.1). Similar data was obtained by the binning method except that the DE genes were now in bins labelled OR and Only, which reduced the large list of DE genes into manageable quanta. Moreover, the inherent value of the bins is inferred transcriptional kinetics. However, the binning method has a cost, which is reflected in the few statistically significant biological functions enriched by DAVID analysis. In contrast, the DE-based method generates a large list, which requires the investigator to decide the fold-change that might be relevant for his/her study. The one advantage of the DE method is that it produces a wide range of functional enrichments (12 GO terms) for DE gene-list for DAVID analysis (Additional file 7: Table S6.3).

Next, we analysed temporal comparison between P0 vs. P21 *Nrl*-WT and P0 vs.P21 *Nrl*-KO by both the binning method and the DE-based method (Additional file 6: Table S5.2, S5.3). Here the binning method revealed gene transcription kinetics across time, which is inherently valuable for deciphering developmental changes in normal and aberrant situations. The DE-based method produced a large DE gene-list, but did not yield any change in gene transcription over time (Additional file 7: Table S6.1, S6.2). Thus, it would require further deconstruction of the list, which is not necessary by the binning strategy. Also, DAVID analysis of the binned data provided enrichment of biological functions tethered to the specific gene expression profile. This is of great value as it is one of the central goals of performing RNA-Seq to capture transcriptome change over time (Additional file 3: Table S2.3, S2.4) . In all, the two approaches of RNA-Seq analysis have merits that can be leveraged to effectively analyse RNA-Seq data.

## Discussion

Amongst the co-transcriptionally regulated genes identified by our binning strategy, genes that remained transcriptionally unaltered employed a higher degree of alternative splicing than those undergoing dynamic regulation. This suggests that during development the major transcription initiations and terminations might lay down the foundation, while the proteome diversity generated through alternative splicing might be engaged in resolving the finer details, such as neuronal subtype specification and terminal differentiation. Another advantage of the binning strategy was that the large transcriptome data was quantized, allowing us to interrogate the dataset for genes with established expression kinetics. The purpose of this was to challenge the binned data generated based on the 1 FPKM threshold for known gene expression patterns (Fig. 3a-b).

Once the dataset was validated, the status of genes in various bins in the E16CE-P0CE comparison was interrogated in the P0CE-P0NE comparison, which revealed dynamic transitions in the transcription kinetics in the same developmental stage. For example, the 632 genes in E16CE_only bin in the E16CE-P0CE comparison bifurcate into No_Ex and P0NE-Only bins in the P0CE-P0NE comparison (Fig. 3e). This suggests that while some genes will remain off, some are re-initiated and predicts that these transcripts will appear in the CE of the next developmental stage. This was confirmed by our quantitative PCR analysis for *Nr2e3, Nrl* and *Rho* across postnatal retinal development [2]. The presence of transcripts for 1084 protein coding genes in the P0NE_Only bin suggested that we had captured *de novo* transcription of genes that might be required for the next developmental program. For example, *Ces5a* is a ~36 kb gene that has FPKM below threshold in E16CE and P0CE, but has FPKM of 161 in P0NE. This value is much higher than FPKM of genes such as *Nr2e3* (35.4 FPKM), *Nrl* (6.1 FPKM) and *Gngt2* (22 FPKM) (Table 5). This indicates that expression of *Ces5a* is more than physiologically equivalent, yet it is not observed in P0CE where it could be translated. One possibility is that there is an active regulation of its export, although this warrants further investigation.

**Table 5:** An example of a “P0NE_Only” gene, Ces5a, whose FPKM unit in P0NE is comparable to those of other genes with known established expression kinetics (*Nrl, Nr2e3, Gngt2*)

Genes	E16CE FPKM	P0CE FPKM	P0NE FPKM
Ces5a	0.073539	0.420057	161.0296
Nrl	0.558403	15.88869	6.104338
Nr2e3	0.008781	28.08	35.45415
Gngt2	10.06518	91.92727	22.08052

The intrinsic value of identifying co-transcriptionally regulated genes is the expectation that they might reveal the biological processes being executed by the developing retina. Our bioinformatics pipeline can deconstruct the order of activation of specific genes engaged in executing a specific biological process so that one can begin to generate gene regulatory networks underlying retinal development. A key feature of our pipeline is the use of GeneMANIA to find potential partners of the core set of genes from a specific bin that enrich for a function in our DAVID analysis (Fig. 2c). A priori, one would predict a progressive increase in the number of genes with sequential application of the GeneMANIA part of the pipeline (Fig. 2c). However, we observed that there was quick convergence in the number of partner genes (Fig. 2c, Right). This suggests that leveraging RNA-Seq data to remove genes that were not expressed in the retina enriched for those genes relevant to retinal development and function at the time point under investigation.

Next we applied our analysis pipeline to find co-transcriptionally regulated genes in the P0 and P21-*Nrl*-WT comparison and the P0 and P21-*Nrl*-KO comparison (Additional file 1: Figure S4, Additional file 5: Table S4). One of the salient features of this analysis was that temporal analysis was more informative than static comparison. One explanation is that temporal analysis created bins that were developmentally regulated, which through DAVID analysis revealed changes in biological processes. For example, there is no cell cycle occurring at P21 so the majority of the cell cycle genes should be inactivated. Indeed, we observe cell cycle in the P0_Only bin in both P0 vs P21-*Nrl*-WT and P0 vs. P21-*Nrl*-KO analysis (Additional file 3: Table S2.3, S2.4). These genes in static analysis would show up as not expressed. Similarly, genes in P21_Only bin enriched for functions such as ion channel activity, ion transport, visual perception, synapse, voltage-gated ion channel activity, neurotransmission and others (Additional file 3: Table S2.3, S2.4). This was as expected as the retina is fully functional at P21 compared to P0. The advantage of our strategy is that it allowed us to understand the progression in gene expression kinetics in normal development and leverage that to understand how this progression deviates in the knockout retina. When P21_Only bin (either P21WT_Only or P21KO_Only) was analyzed through DAVID, we found many functions that were common to both sets of comparison, except examination of the number of genes underlying these functions revealed that there were subtle differences between the two bins (P21WT_only and P21KO_Only) (Additional file 3: Table S2.3, S2.4). This suggested that while many of the functions remain unaltered in the KO, there are subtle changes in the manner in which they might be executed. For example, “visual perception” showed up in the P0 vs. P21-Nrl-WT and P0 vs. P21-Nrl-KO comparisons in the P21_Only bins (Fig. 6). There were 24 genes underlying enrichment of this function in the WT comparison (Fig. 6), while there were 22 genes in KO comparison (Fig. 6). Upon comparing the gene identities from both sets, subtle differences emerged that allowed us to find the biological meaning from change in a single gene such as the rod photoreceptor-specific gene, *Gnat1*, that was absent in the *Nrl*-KO retina, which lack rod photoreceptors [48]. Finding *Gnat1* through temporal analysis raises the question whether it would have been found in static analysis. While *Gnat1* was present in the P21WT_Only bin in static analysis, the rest of the genes that would normally be part of the GO term “visual perception” were in the Non_DR bin. Thus, without a priori knowledge one would not find this specific gene out of the entire list of genes in the P21WT_Only. Temporal analysis combined with our gene expression and binning strategy followed by our custom bioinformatics pipeline was able to find these subtle changes, which in case of static analysis was not possible (Additional file 1: Figure S4). While one could find these subtle changes in the static analysis by looking at specific genes, it requires a priori knowledge. The advantage of doing whole transcriptome analysis is that one could find patterns computationally, which can be leveraged to obtain new insights without the need for a priori knowledge. For example, GO terms such as potassium channel complex, sodium channel activity, synaptic vesicle, calcium ion transport and regulation of blood pressure regulation (Additional file 3: Table S2.4) were enriched by genes in the P21_Only bin in P0 vs. P21-*Nrl*-KO comparison, but were absent in the WT comparison. This finding suggests that there are specific anomalies in the *Nrl*-KO retina. Given that in the *Nrl*-KO retina, the majority of the rod photoreceptors have converted to cone photoreceptors, changes in ion transport and synaptogenesis are to be expected [53, 54]. However, regulation of blood pressure seemed out of place for the *Nrl*-KO retina. Indeed, closer examination of the genes underlying this function revealed the need to examine vasodilation in the *Nrl*-KO retina. Notably, previous reports showed that the *Nrl*-KO retina develops dilated retinal blood vessels and leakage at P60 [52]. Thus, this confirmed the prediction made through shifts in the molecular signatures identified by our temporal analysis. Importantly, our analysis predicted an outcome based on gene expression pattern changes occurring between P0 to P21 that manifests at P60.

## Conclusions

In summary, we were able to extract shifts in biological processes (Fig. 4a) governed by precise changes in gene expression through our unique RNA-Seq data acquisition/analysis platform. Importantly, it showed that the nuclear transcriptome was temporally shifted ahead of the cytoplasmic transcriptome at a developmental timepoint (Fig. 4c). Overlapping these discoveries with those made by extending our strategy to P21-*Nrl*-WT and KO analysis was most fruitful when ∆/time was extracted. This strategy identified perturbation in the molecular signature that enabled prediction of a phenotype that would manifest histologically at a later time (Fig. 4b). Indeed, this strategy would be effective toward deconstructing the progression of molecular changes during aberrant development or the progression of pathogenesis of the retinal diseases and can be extended to other tissues.

## Additional files

Additional file 1: Supplemental Figures. (PDF 1143 kb) Additional file 2: Table S1. Output of Binning in E16CE – P0CE and P0CE –P0NE comparisons. Custom bioinformatics pipeline and binning of E16CE - P0CE and P0CE-P0NE comparisons at the gene level (S1.1, S1.3) and isoform level (S1.2, S1.4) as discussed in the strategy in Fig. 1c. (XLSX 10709 kb) Additional file 3: Table S2. DAVID analysis output for static and temporal comparisons. DAVID output for genes belonging to bins in E16CE-P0CE comparison (S2.1), P0CE-P0NE comparison (S2.2), P0 vs. P21-*Nrl*-WT comparison (S2.3),P0 vs. P21-*Nrl*-KO comparison (S2.4) and P21-*Nrl*-WT vs. P21-*Nrl*-KO comparison (S2.5). (XLSX 140 kb) Additional file 4: Table S3. Microarray analysis results. Clusters generated through K-means clustering in Genesis for the microarray. (XLSX 330 kb) Additional file 5: Table S4. Binning results in P21-*Nrl*-WT vs. P21-*Nrl*-KO, P0 vs. P21-*Nrl*-WT and P0 vs. P21-*Nrl*-KO comparisons. Custom bioinformatics pipeline and binning of P21-*Nrl*-WT vs. P21-*Nrl*-KO (S4.1), P0 (P0CE + P0NE) vs. P21WTcomparison (S4.2) and P0 (P0CE + P0NE) vs. P21KO comparison (S4.3). (XLSX 4018 kb) Additional file 6: Table S5. DE based analysis results for static and temporal comparisons. DE based analyses for static (P21WT vs. P21KO) (S5.1) and temporal (P0 vs. P21WT and P0 vs. P21KO) comparisons (S5.2, S5.3). (XLSX 4196 kb) Additional file 7: Table S6. DAVID analysis output of DE based analyses for static and temporal comparisons. Results of DAVID analysis based on DE genes for P0 vs. P21WT (S6.1), P0 vs. P21KO (S6.2), and P21WT vs. P21KO (S6.3) comparisons. (XLSX 41 kb)
