# Supplementary material for: Network-based bioinformatics analysis of spatio-temporal RNA-Seq data reveals transcriptional programs underpinning normal and aberrant retinal development
Source: BMC Genomics. 2016 Aug 31;17(Suppl 5):495. doi: 10.1186/s12864-016-2822-z (PMC5009874; doi:10.1186/s12864-016-2822-z)
Supplement: Additional file 1: — Supplemental Figures. (PDF 1143 kb) [file 12864_2016_2822_MOESM1_ESM.pdf]

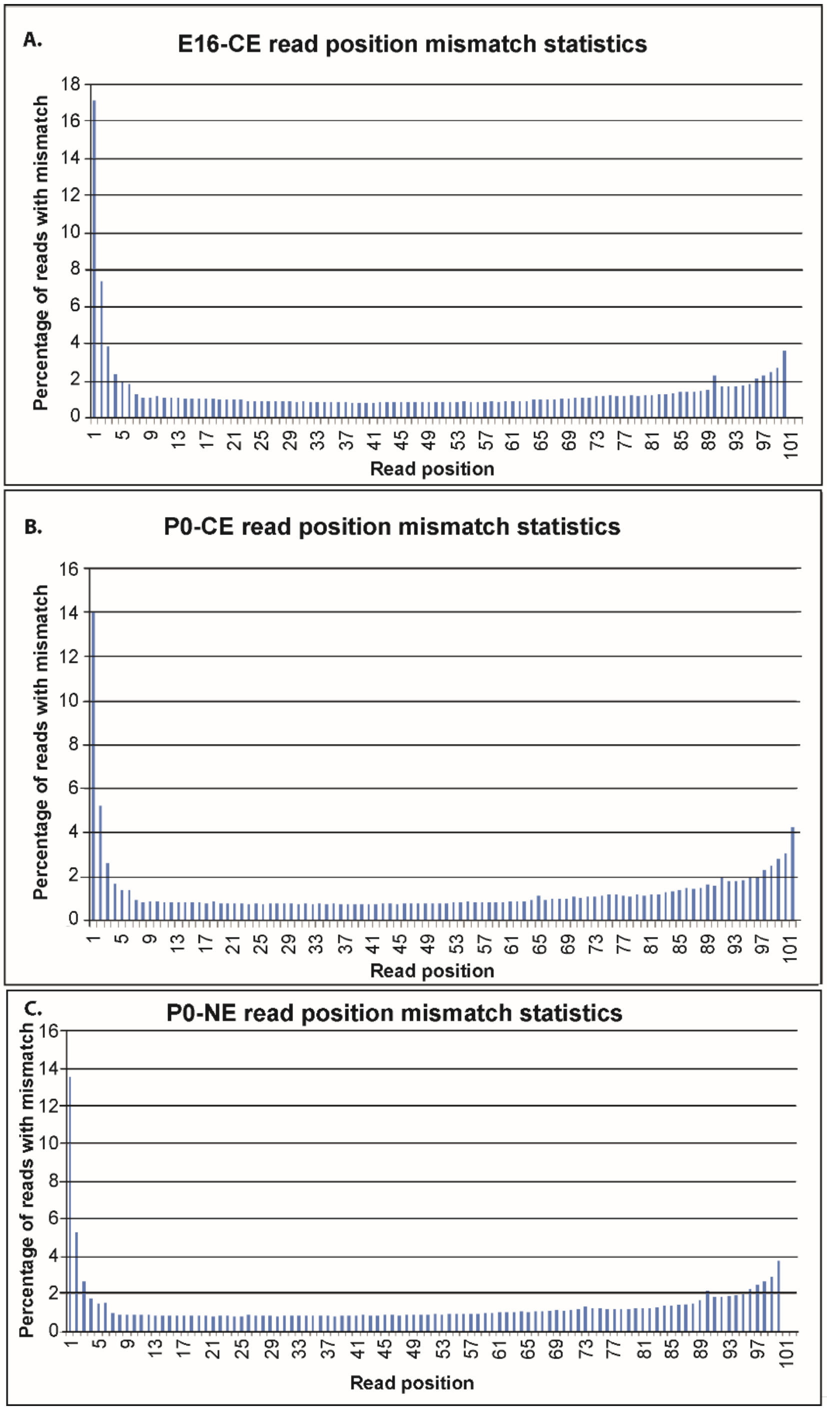

Figure S1 (Related to Materials and Methods): Read position mismatch analysis. A – C. Reads obtained from deep RNA sequencing for E16CE (A), P0CE (B) and P0NE (C) were subjected to mismatch analysis by HardMerge alignment. Shown in x-axis is the read position (1 – 101) and shown in y-axis is the percentage of reads with mismatch.

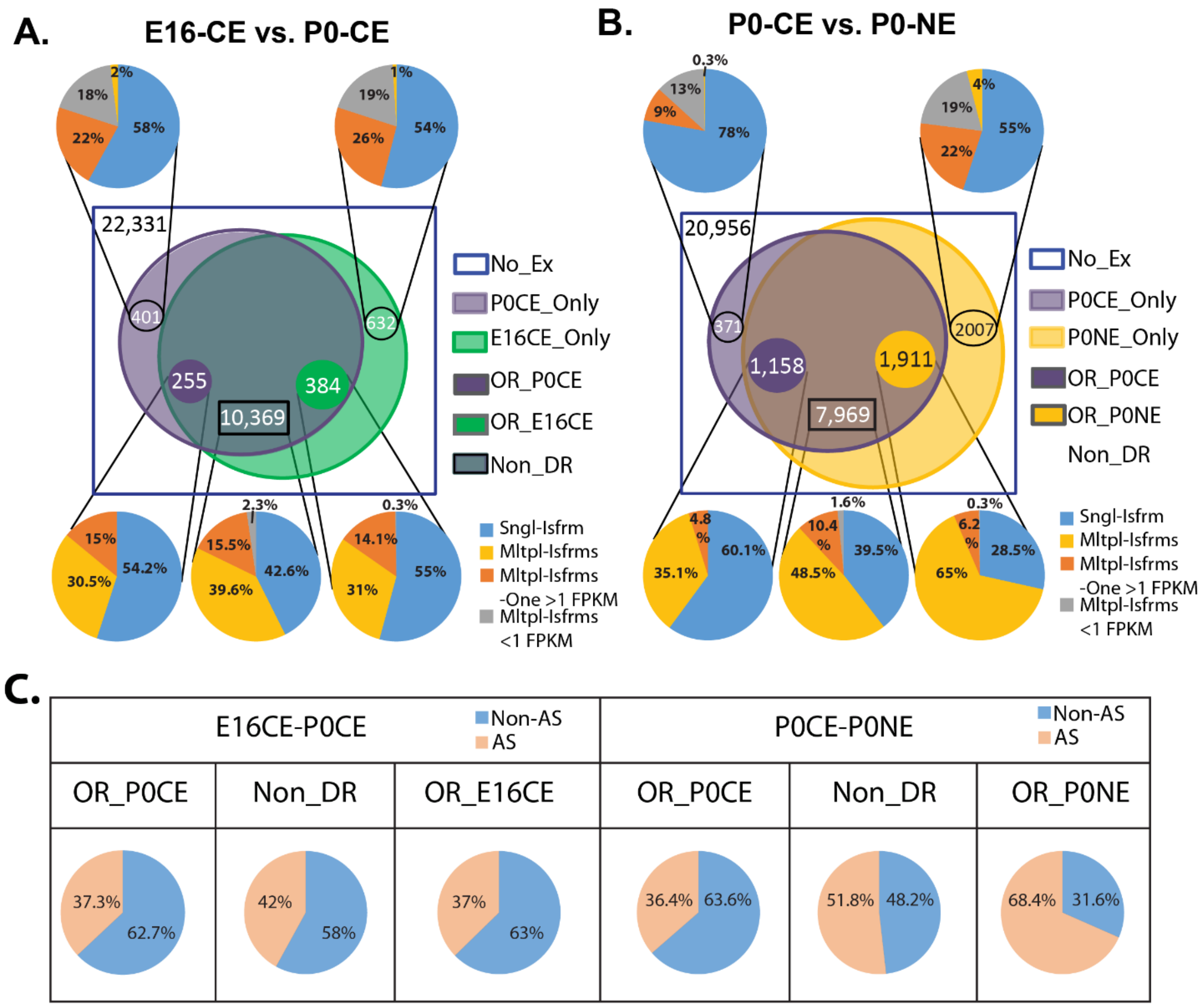

Figure S2 (Related to Fig. 2): Transcriptome summary for E16CE-P0CE & P0CE-P0NE comparison: A & B. Transcriptome summary of the comparison between E16CE-P0CE and P0CE-P0NE, respectively. The white box represents all genes examined for this study. The venn diagram within it represents the different bins. **C.** Alternative splicing (AS) status of “expressed in both” groups (OR_E16CE, Non_DR, OR_P0CE, and OR_P0NE) in E16CE-P0CE and P0CE-P0NE comparisons.

A.

		P0CE - P0NE			
		Non_DR	OR_P0CE	OR_P0NE	P0NE_Only
E16CE - P0CE	E16CE_Only	-	-	-	*Dtna, Drp2*
	Non_DR	*Cyfip2, Gria1, Cadps, Rims2, Dlg4, Dlgap3, Nde1, Atp2b2, Cplx1, Dlgap4, Nit1, Sdcbp, Ndel1, Dlgap5, Dlg1, Grip1, Unc13a, Sipa1l1, Dlg3, Cask, Fam49a, Fam49b, Snta1*	-	*Cadps2, Dmxl2, Ank3, F2r, Camk2d, Cplx2, Cyfip1, Dtnb, Dmd, Gria2, Gria4, Grik2, Grm7, Grm8, Gad2, Itpr1, Lrrk2, Magi2, Mpdz, Myo6, Myo7a, Myh10, Myh9, Nrxn1, Nrxn3, Nbea, Nlgn1, Ophn1, Plcb4, Psd3, Dennd1a, Rab3c, Slc1a2, Sos1, Sypl, Syt1, Tanc1, Unc13b, Unc13c, Utrn, Kif1b, Zdhhc17*	-
	OR_E16CE	*Syt13*	-	*Agrn, Snbt2*	-
	OR_P0CE	-	*Cplx3*	*Dlg2*	-
	P0CE_Only	*Grin1, Grm3*	-	*Dlgap1, Gria3, Gad1, Kcnma1*	-
	No_Ex	-	-	-	*Grid2, Ryr2, Shank2, Grik5, Grin2b, Grid1, Grin3a*

B.

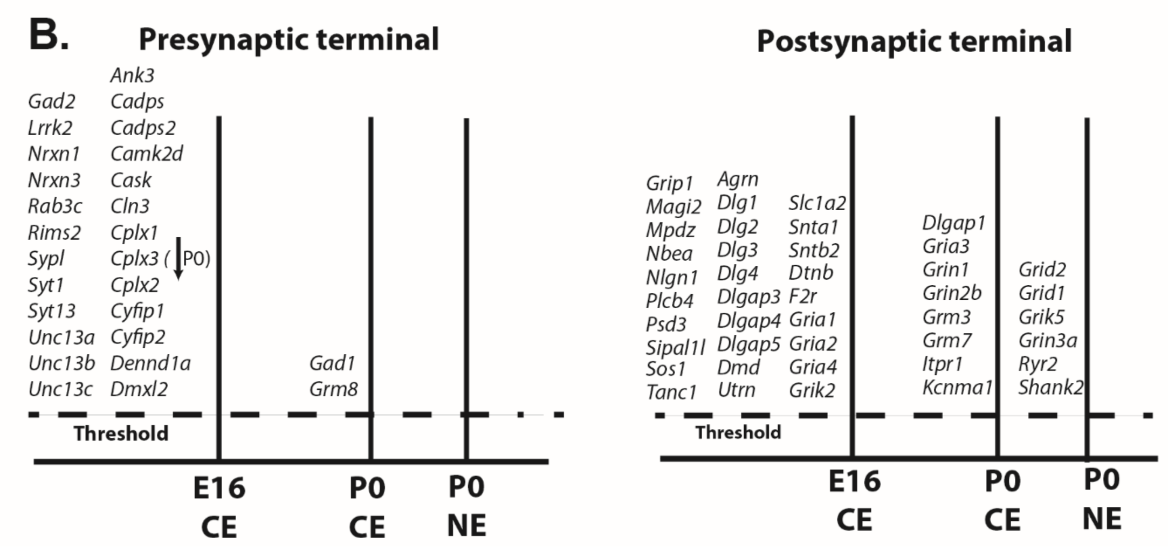

Figure S3 (Related to Fig. 4): Downstream analysis of genes belonging to the OR_P0NE bin enriching for the GOterm "Synapse": A. Output of the downstream gene annotation analysis pipeline parts II and III for OR_P0NE category which enriched for synapse. **B.** Genes resulted

from the DAVID analysis for the GOterm “synapse” were further sub-divided into presynaptic or postsynaptic along with the time point of their transcription initiation. Up (↑) and down (↓) arrows indicate upregulation or downregulation of transcription at that time point.

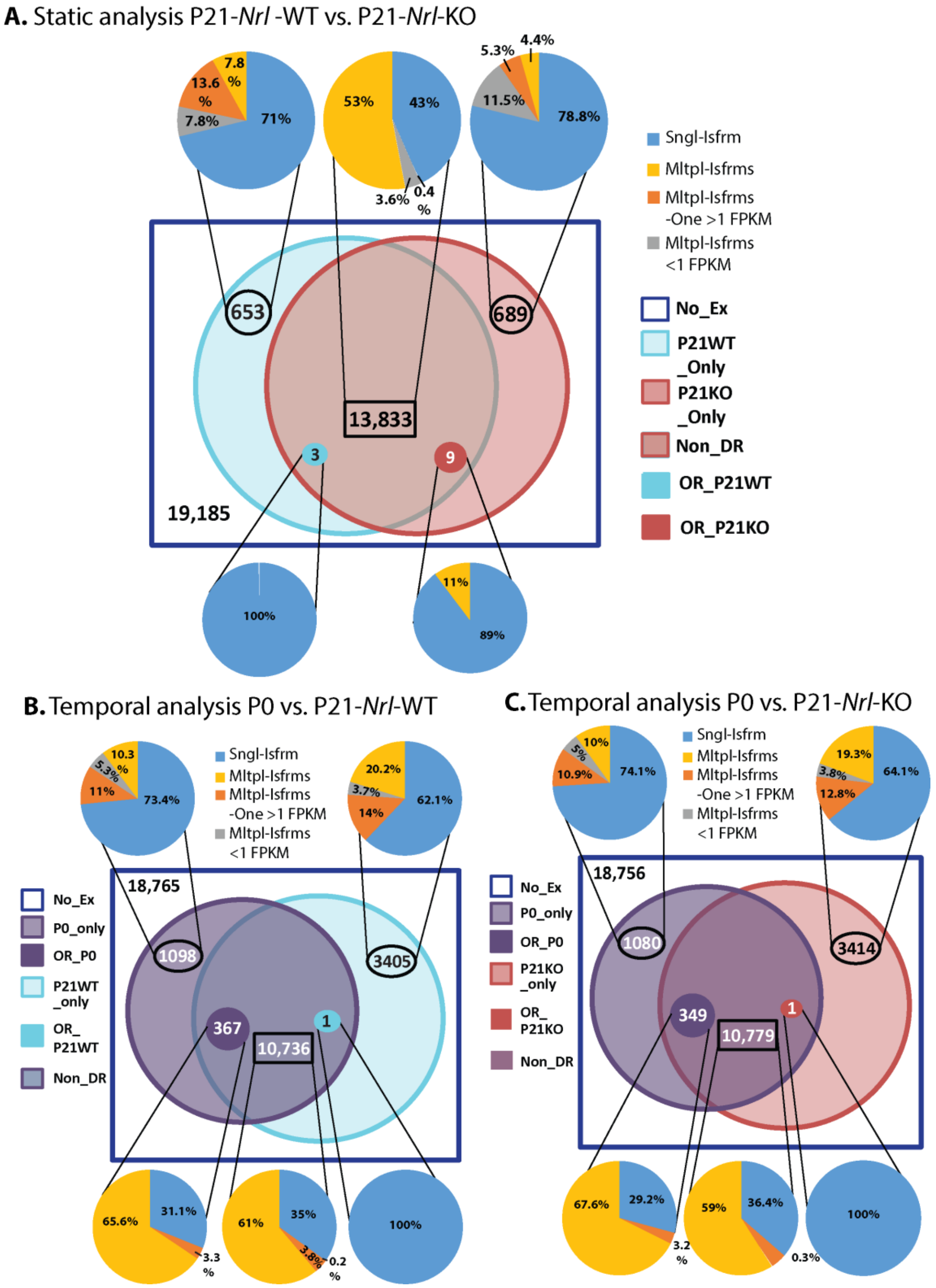

Figure S4 (Related to Fig. 5): Transcriptome summary for static & temporal comparison: A. Transcriptome summary of the comparison between P21-*Nrl*-WT and P21-*Nrl*-KO (Static comparison). The white box represents all genes examined for this study. The venn diagram within it represents the different bins. **B & C.** Transcriptome summary of the comparison

between P0 vs. P21-*Nrl*-WT and P0 vs. P21-*Nrl*-KO, respectively (Temporal comparison). The white box represents all genes examined for this study. The venn diagram within it represents the different bins.
